# 11th Asia Oceania Human Proteome Organization Congress Report

**DOI:** 10.1016/j.mcpro.2023.100627

**Published:** 2023-08-01

**Authors:** Angus C. Grey, Qingsong Lin, Teck Yew Low, Wei Wu, Paul A. Haynes, Maxey C.M. Chung, Yu-Ju Chen, Stuart J. Cordwell, Yasushi Ishihama, Ping Xu, Peter Hoffmann, Ho Jeong Kwon, Terence C.W. Poon

**Affiliations:** 1 Department of Physiology, University of Auckland, Auckland, New Zealand; 2 Department of Biological Sciences, National University of Singapore, Singapore; 3 UKM Medical Molecular Biology Institute (UMBI), Universiti Kebangsaan Malaysia, Kuala Lumpur, Malaysia; 4 Singapore Immunology Network (SIgN), Agency for Science, Technology and Research (A∗STAR), and Department of Pharmacy, National University of Singapore, Singapore; 5 School of Natural Sciences, Macquarie University, North Ryde, Nova Scotia, Australia; 6 Department of Biochemistry, Yong Loo Lin School of Medicine, National University of Singapore, Singapore; 7 Institute of Chemistry, Academia Sinica, Taipei, Taiwan; 8 School of Life and Environmental Sciences and Sydney Mass Spectrometry, The University of Sydney, Sydney, Australia; 9 Graduate School of Pharmaceutical Sciences, Kyoto University, Kyoto, Japan; 10 State Key Laboratory of Proteomics, Beijing Proteome Research Center, National Center for Protein Sciences (Beijing), Research Unit of Proteomics & Research and Development of New Drug of Chinese Academy of Medical Sciences, Beijing Institute of Lifeomics, Beijing, China; 11 Clinical and Health Sciences, University of South Australia, Adelaide, South Australia, Australia; 12 Chemical Genomics Leader Research Initiative, Department of Biotechnology, Yonsei University, Seoul, South Korea; 13 Pilot Laboratory, Proteomics Core, Institute of Translational Medicine, Centre for Precision Medicine Research and Training, Faculty of Health Sciences, University of Macau, Macau SAR, China

**Keywords:** AOHUPO, proteomics, Asia-Oceania

## Abstract

As the first in-person Asia Oceania Human Proteomics Organization (AOHUPO) congress since 2018, the 11th AOHUPO congress was an opportune time for the research community to reconnect and to renew friendships after the long period of restricted travel due to the global pandemic. Moreover, this congress was a great opportunity for the many AO regional proteomics and mass spectrometry scientists to meet in Singapore to exchange ideas and to present their latest findings. Cohosted by the Singapore Society for Mass Spectrometry and the Malaysian Proteomics Society and held in conjunction with the seventh Asia Oceania Agricultural Proteomics Organization Congress and Singapore Society for Mass Spectrometry 2023, the meeting featured both human and agricultural proteomics. Over five hundred scientists from the AO region converged on the MAX Atria @ Singapore EXPO, Changi, Singapore from May 8 to 10 for the main congress. The diverse program was made up of 64 invited speakers and panellists for seven plenary lectures, 27 concurrent symposia, precongress and postcongress workshops, and 174 poster presentations. The AOHUPO society were able to celebrate not only their 20th anniversary but also the outstanding academic research from biological and agricultural proteomics and related ‘omics fields being conducted across the Asia-Oceania region.

## Highlights

• Two scientific themes on human and agricultural proteomics, and mass spectrometry techniques.
• 64 invited speakers for seven plenary lectures, 27 symposia, and associated workshops.
• One hundred seventy four posters presented in networking sessions over 3 days.
• Integration of academic research from biomedical and agricultural fields.

## Graphical Abstract

**Figure:**
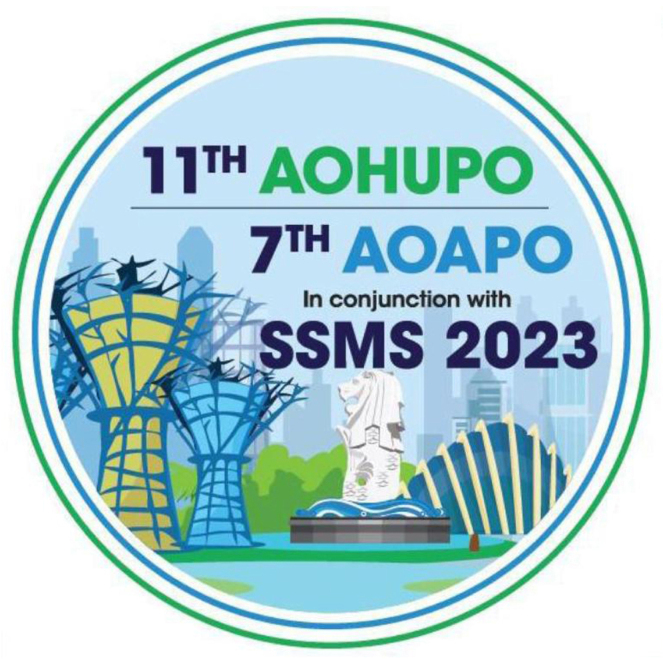

## In Brief

Report on 11th Asia Oceania Human Proteome Organization Congress in Singapore.

**Fig. 1:**
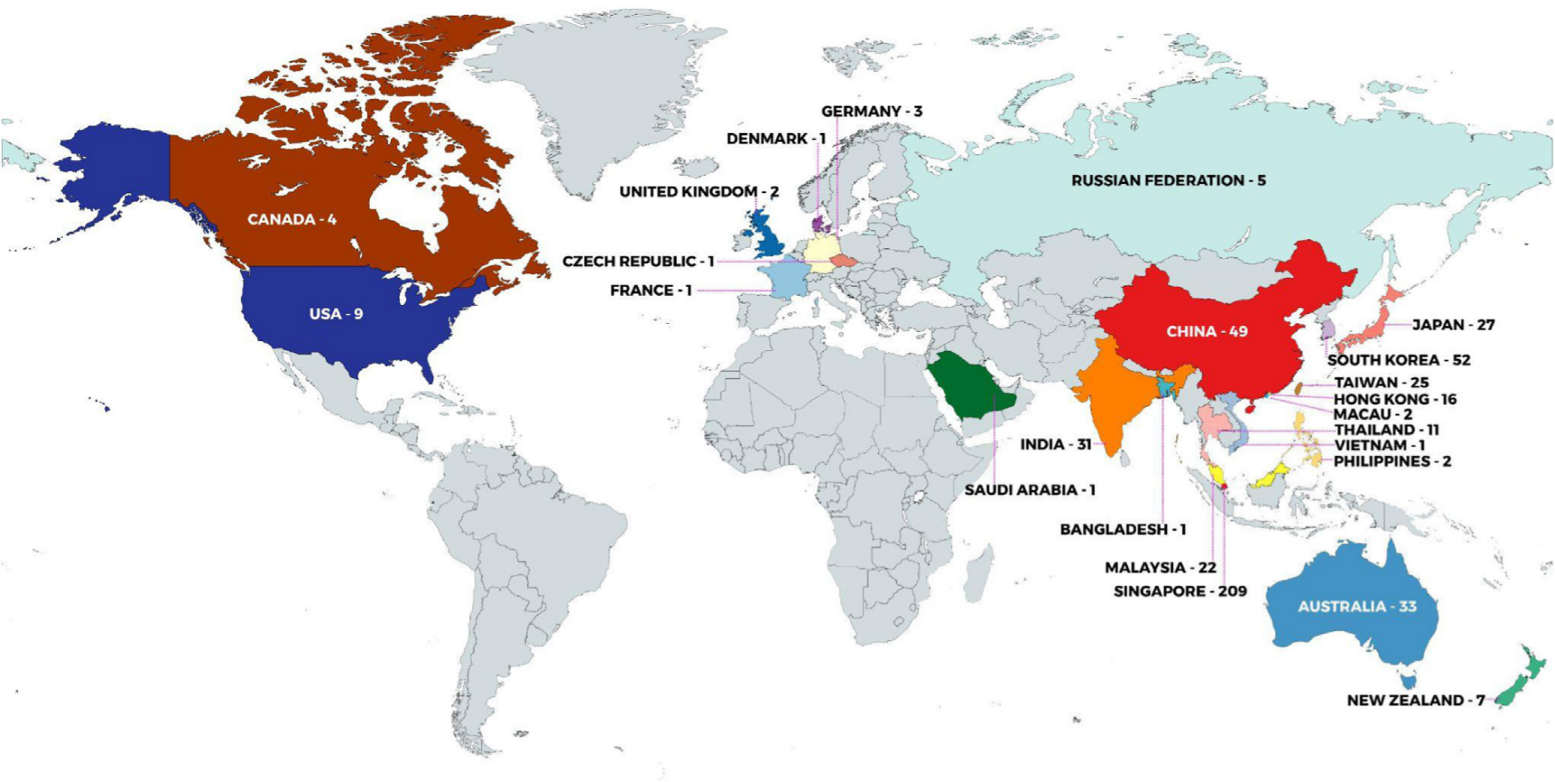
**World map showing registration statistics for the congress**.

The Asia Oceania Human Proteome Organization (AOHUPO) was established in June 2002 through the efforts of the late Akira Tsugita (Japan), Richard Simpson (Australia), Young-Ki Paik (Korea), and Kazuyuki Nakamura (Japan). The main aim of AOHUPO is to promote and coordinate the activities of the regional proteomics community, and with this in mind, it has firmly established the tradition of organizing a highly successful biennial conference being hosted by different countries in the region. Since the first meeting in Seoul, Korea, in 2002, the congress has gone from strength to strength, and the 11th edition of the congress was held in Singapore from May 8 to 10. AOHUPO 2023 was the first in-person congress following the Covid-19 pandemic, which disrupted global travel from early 2020 to late 2022. In addition, the congress was the first combined Asia Oceania organization congress, having been jointly held with the seventh Asia Oceania Agricultural Proteomics Organization (AOAPO) meeting. Furthermore, it was the first AOHUPO congress co-organized by two regional societies, the Singapore Society of Mass Spectrometry and the Malaysian Proteomics Society. Over five hundred scientists from the Asia Oceania (AO) region converged on the MAX Atria @ Singapore EXPO, Changi, Singapore for the main congress. The diverse program was made up of 64 invited speakers and panelists for seven plenary lectures, 27 concurrent symposia, precongress and postcongress workshops, and 174 poster presentations. In the following report, we share the congress highlights to recognize and celebrate all of the contributions that made the congress so successful. With 515 delegates from 22 countries, the 11th AOHUPO meeting truly reflected the diversity and quality of proteomic and mass spectrometry–related research being conducted across the AO region (Fig. 1).

## Precongress Workshop (May 7th 2023)

**Fig. 2:**
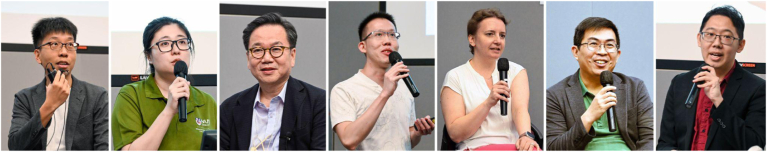
**Invited speakers for the precongress workshop.** Held at the National University Singapore, featuring career outlook talks from (*left* to *right*) Warren Kok Yong Tan (SGInnovate), Wei Wu (National University of Singapore and A∗STAR, Singapore), Ho Jeong Kwon (Yonsei University, Seoul, South Korea), Lian Jie Bay (Singapore Food Agency Research Scientist), Hanna Budayeva (Genentech, Chemical Proteomics Division Lead), Bin Zou (CEO, Axcynsis Therapeutics), and Xin Xiang Lim (National University of Singapore).

The main congress was prefaced by a full-day workshop that was themed as a Young Scientist Forum (YSF). The YSF was an informal platform that encouraged interaction and scientific exchange among young talented researchers in the Asia-Oceania region. This one-day event was attended by 86 researchers and featured four sessions for selected young scientists who had received “Young Scientists Travel Awards” to showcase their cutting-edge research on the applications of proteomics and mass spectrometry (MS) in therapeutics development, biomarker discovery, environment & sustainability, as well as structural biology. Besides scientific talks, the YSF program was interspersed with a career development forum, where YSF advisors came from a venture capitalist firm, Singapore Agency for Science, Technology and Research (A∗STAR), Singapore Food Agency, Yonsei University, Genentech and Axcynsis Therapeutics shared their diverse personal experiences and tips on their career progression as young and senior principal investigators, venture capitalist, and scientists in the industry and government agency (Fig. 2).

The YSF was hugely successful, with comments from student attendees including Alyssa Leong (YSF Travel Award winner from Malaysia), who wrote, “I’m honoured for the opportunity to showcase my work at such a prestigious conference such as the AOHUPO Congress. This experience has been eye-opening for me towards the field of proteomics, the current work being done from different angles to advance human knowledge is inspiring and impressive. As a YSF Award recipient, I am also grateful for the career panels and the opportunity to connect with so many people across the globe. I am looking forward to the next AOHUPO and hoping that I will be a part of it too.”

In addition, Catherine Maidment, a PhD student from New Zealand who attended the YSF wrote, “I attended the young scientist forum at the National University of Singapore on the 7^th^ of May. This gave me an opportunity to interact with young scientists, especially PhD students, before the conference and listen to their research. Scattered throughout the day were career talks from different areas including a venture capitalist, principal investigator, research scientist and lecturer which gave us an opportunity to learn about the everyday life and advantages and disadvantages of each position and strengthened my desire to continue as a researcher after completing my PhD. Overall it was a very informative and enjoyable day.”

## Day 1 (May 8th 2023)

The first day of the congress featured three plenary sessions, nine concurrent symposia, and a special evening session to celebrate the 20th anniversary of the formation of AOHUPO. In the morning session, all delegates were officially welcomed to the conference by co-chairs Prof Qingsong Lin (SMSS, National University of Singapore) and Prof Teck Yew Low (Malaysian Proteomics Society, Universiti Kebangsaan Malaysia). Prof Lin introduced the main scientific themes for the conference, which included biological, biomedical, clinical, agricultural, environmental, and aquatic proteomics, with additional mass spectrometry–related ‘omics, applications and technologies also included. On behalf of AOHUPO, the current president Prof Terence Poon (University of Macau) also welcomed delegates and highlighted the strong links between the conference’s contributing societies and organizations. He noted that through advancements in agricultural and biomedical proteomics, we can all contribute to the improvement of human health on a global scale. Finally, Prof Paul A. Haynes (Macquarie University), president of the AOAPO, welcomed all delegates. He noted the impacts of the pandemic period on research progress and our ability to meet and discuss our research and ideas. He highlighted the strengths of in-person events and encouraged us all to make the most of this cross-cultural, cross-discipline meeting.

The scientific program began with excellent plenary presentations in the morning. The first was from Prof Mathias Wilhelm (Technical University Munich) on developing bioinformatic tools using deep learning strategies to better identify and assign tandem MS spectra from data-independent analysis data sets. In the second, Prof Fuchu He (National Center for Protein Sciences Beijing) compared the current state of proteomics to the more advanced genomics field and stressed the importance of further developing proteomics approaches to fully characterize the human proteome and apply this knowledge in precision medicine applications. He presented his grand vision for a human proteomics resource, π-HuB (Proteomic Navigator of the Human Body) (https://www.pi-hub.org.cn/), and the initial steps that have been achieved in this multidecade research plan.

**Fig. 3:**
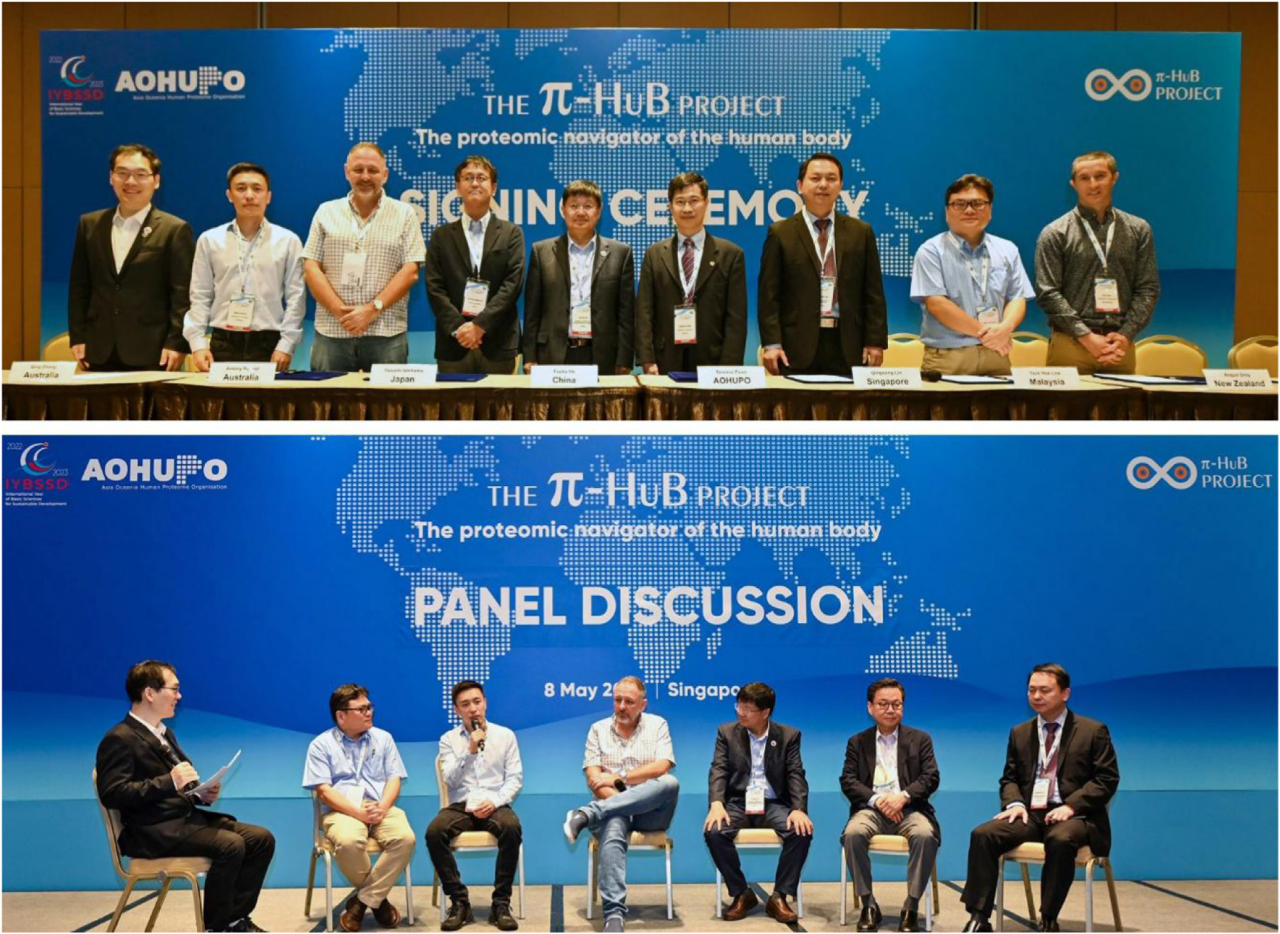
**Events to mark the collaboration between AOHUPO and the π-HuB project.** Signing ceremony (*above*) and members of the panel discussion (*below*). AOHUPO, Asia Oceania Human Proteomics Organization.

Following Prof He’s plenary lecture, a ceremony was held to mark the signing of a memorandum of understanding between AOHUPO and π-HuB. Prof He (π-HuB) and Prof Poon (AOHUPO) signed on behalf of their organizations, which was witnessed by several AOHUPO council members from across the AO region (Fig. 3, *upper*). Next, a panel discussion was held to explore the vision and work plan for π-HuB, which included excellent contributions from the panel (Fig. 3, *lower*) on topics ranging from data sovereignty and protocol standardization to data integration. Following this, the first draft of the white paper on the π-HuB project will be circulated for further discussion and improvement. There was general agreement that the π-HuB project is ambitious and exciting. A postcongress workshop was also held immediately after the conference closed.

The concurrent congress sessions on day 1 included the themes metabolomics, biomarker discovery and disease proteomics, and drug discovery. Following the regular sessions, attendees enjoyed a plenary presentation from Prof Markus Wenk (National University of Singapore), who spoke about lipidomic profiling in humans. He highlighted his work investigating ceramides, their association with adverse cardiovascular events, and the modification of their circulating levels by either lifestyle or pharmaceutical interventions.

**Fig. 4:**
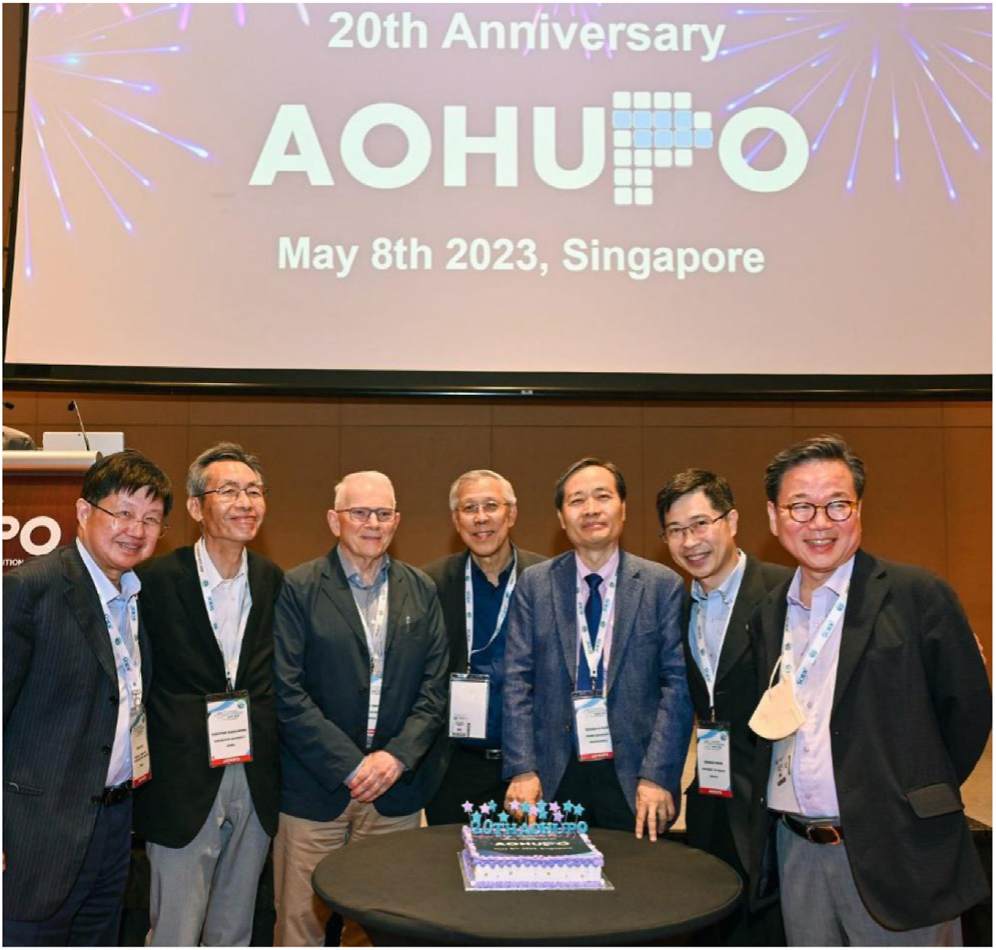
**20th anniversary celebration for AOHUPO.** Former and current AOHUPO presidents assembled as a cake to celebrate 20 years since the formation of AOHUPO was cut. Pictured (*left to right*) are Fuchu He, Kazuyuki Nakamura, Richard Simpson, Maxey Chung, Young-ki Paik, Terence Poon, and Ho Jeong Kwon. AOHUPO, Asia Oceania Human Proteomics Organization.

The final session on congress day 1 was dedicated to celebrating the 20th anniversary of the formation of AOHUPO. This in-person celebration was delayed from the scheduled celebration in 2021 when the conference was held online due to travel restrictions as a result of the global pandemic. This was therefore the first in-person opportunity to recognize the anniversary and celebrate how far the society has come in 20 years. Prof Poon welcomed everyone to the session and highlighted how the society had thrived and expanded since its establishment. He expressed his wish to both celebrate the past and look forward to the future. Prof Poon then invited each past president to the stage, presented them with commemorative plaques, and asked them to share their thoughts and memories of AOHUPO. The inaugural president, Prof Richard Simpson, described the research environment in the lead up to AOHUPO and the factors that drove its formation. He noted just how far the field has progressed, since he recalled protein analysis *via* Edman sequencing and a bioinformatics approach that took more than a month and involved mail correspondence with Prof Margaret Dayhoff. Finally, he paid tribute to the late Prof Akira Tsugita, who helped found AOHUPO. Next, Prof Young-ki Paik shared pictures and the story of AOHUPO’s formation (June 2002), shortly before KHUPO (July 2002). He noted the democratic vote to determine a suitable logo, which was held at the 2002 KHUPO meeting. Thanks to Prof Paik, all attendees of this event now know that the eight blue dots that appear in the ‘P’ of the logo, which is a feature of many proteomics societies and organizations across the globe, stand for the eight letters in P-R-O-T-E-O-M-E. Our third president, Prof Kazuyuki Nakamura, took the opportunity to give a short presentation on a clinical proteomics project which was personally meaningful. His talk demonstrated the progress and further work required on plasma protein biomarkers of Alzheimer's disease to allow earlier detection and potential cures. Prof Fuchu He then shared his ‘Dream trip with AOHUPO’. He described how AOHUPO was a blueprint for HUPO and thanked all of the past presidents for providing an excellent blueprint for his presidency of AOHUPO. He also described the establishment of CNHUPO in 2002 (October), which was clearly a busy year for the global proteomics community. Prof Maxey Chung then described his personal proteomics history as a council member of AOHUPO for 20 years and his long association with HUPO. His organization of the third AOHUPO congress (December 2006) in Singapore was a personal highlight, and he shared photos of many colleagues at previous meetings who were present at the celebration. Finally, he acknowledged all of the next generation of proteomics researchers who were involved in organizing this 11th AOHUPO. Our immediate past president Prof Ho Jeong Kwon then remarked that his presidency began as the pandemic hit, and he feared for how that would affect AOHUPO. However, Prof Kwon described the energy and desire that AOHUPO has to overcome challenges and how the effects of travel restrictions were overcome by holding the 10th AOHUPO congress and subsequent online education series (OES) (1). Finally, he thanked the members of the executive committee that served during his presidency and noted that this global crisis was also an opportunity to unite, which AOHUPO was able to do successfully. The final acknowledgment of the evening went to Dr Bill Jordan (Victoria University Wellington, NZ). Dr Jordan joined the session online *via* video link and was thanked by Prof Poon for his tremendous support of AOHUPO in its formative years. To recognize this, Prof Poon presented him an Honorary Membership of AOHUPO. Dr Jordan congratulated AOHUPO for its 20th anniversary and encouraged all delegates present to carry on their important research activity and collegiality in the AO region. Following the presentations, all AOHUPO council members, past and present, were invited on stage for cake-cutting and a photo to mark the occasion (Fig. 4). While the session was an opportunity for some to reflect, for others, it was an interesting history lesson and a source of inspiration for future research and collaboration in the AO region.

## Day 2 (May 9th 2023)

The second day of the congress featured three plenary sessions, nine concurrent symposia, and the conference dinner evening event. Topics featured in the concurrent symposia included posttranslational modifications (phospho- and glyco-proteomics, glycomics), drug discovery and clinical applications, proteogenomics and immunopeptidomics. Two morning plenary sessions set the tone for an excellent day of presentations. In the first plenary, Prof Dame Carol Robinson (Oxford University) presented her laboratory’s efforts to determine membrane protein structure and interaction using native mass spectrometry. Her latest method used cell vesicle preparations to maintain membrane protein complexes in their native environment, and this was illustrated using dark and light-adapted rhodopsin. Prof Ho Jeong Kwon (Yonsei University) delivered the secondary morning plenary presentation, which detailed efforts to understand the autophagy process and its role in several major human diseases. Prof Kwon discussed three different naturally occurring compounds and their mechanisms in mitigating pathological autophagic processes in senescence, obesity, and atherosclerosis by label-free target identification of the three compounds using DARTS and LC-MS/MS proteomics methods. Following the concurrent symposium sessions, Prof Yu-Ju Chen (Academia Sinica) delivered the afternoon plenary presentation. Working in clinical proteomics and with a desire to understand lung cancer in nonsmoking patients, Prof Chen discussed the current challenges in shifting from genetic to protein-based diagnostics for cancer. To address some of these challenges, Prof Chen presented novel microfluidics devices to enable single cell proteomics and phosphoproteomics. Her approaches to single cell analysis have been able to detect important cancer pathway proteins that may explain the variability in lung cancer outcomes observed in the patients that she has studied.

The AOAPO research network held a productive lunch time workshop focusing on the ‘Proteomes that feed the world’ initiative being led by Profs Bernhard Kuster and Mathias Wilhelm of the Technical University of Munich. This included highlighting current progress and future plans. It was also noted that there were still some crop species on the list that were yet to be thoroughly investigated and several of those would be good candidates for initial genome sequencing studies.

In the evening, the very popular conference dinner was held at the Dusit Thani Laguna, Singapore. Delegates made the most of the opportunity to catch up with old friends and make new connections over an expansive buffet dinner and dessert menu that was delicious.

## Day 3 (May 10th 2023)

The third day of the congress featured nine concurrent symposia, one plenary session, and the closing ceremony and prize giving. The concurrent symposia featured talks in sessions themed in infectious disease, native mass spectrometry, systems biology, mass spectrometry technologies, and bioinformatics. In the final plenary session, held in the afternoon, Prof Shaojun Dai (Shanghai Normal University) showed how he has used a multi-omics approach to understand the salinity stress response in forage grass. In particular, he showed how the phosphoproteome and cell signaling pathways respond to this stress. Given our changing climate, this was a fitting way to bring the congress scientific program to a close.

**Fig. 5:**
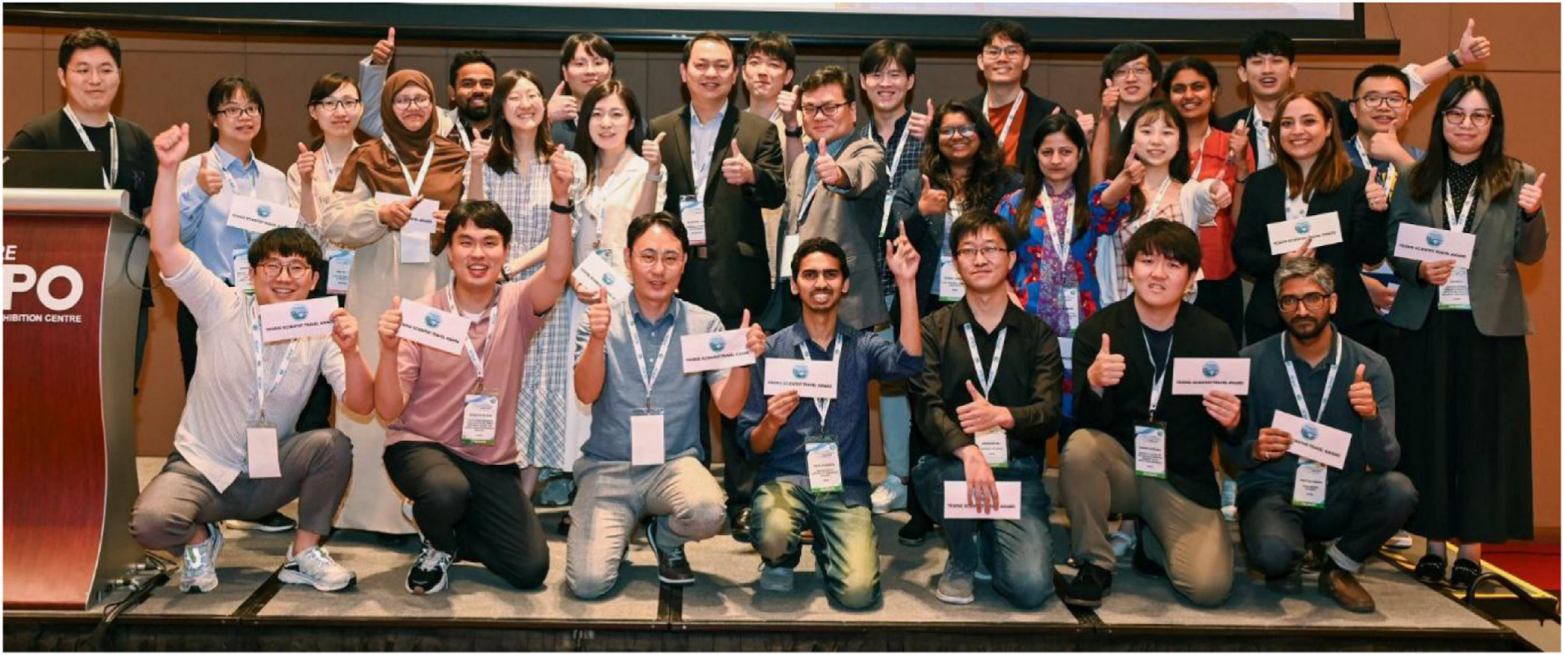
**AOHUPO young scientist travel award winners**. AOHUPO, Asia Oceania Human Proteomics Organization.

The final session was devoted to the presentation of congress awards and official closing. Twenty-eight young scientist awards were presented by congress co-chairs Prof Qingsong Lin and Prof Teck Yew Low to recognize their research potential and encourage them to begin to form their own linkages across the AO region. Students from around the AO region, and beyond to the Netherlands, were recognized (Fig. 5). Following the awards, all delegates were invited to attend the upcoming 22nd Annual HUPO World Congress 2023 (17–21 September 2023 in Busan, Korea; https://2023.hupo.org/) and the 12th CNHUPO Congress & the π-HuB project Global Summit (24–27 September 2023 in Chengdu, China; http://cnhupo.ittn.com.cn/). Prof Low then gave the closing address and thanked past presidents, sponsors, conference organizers, and attendees for making the conference such a resounding success.

**Fig. 6:**
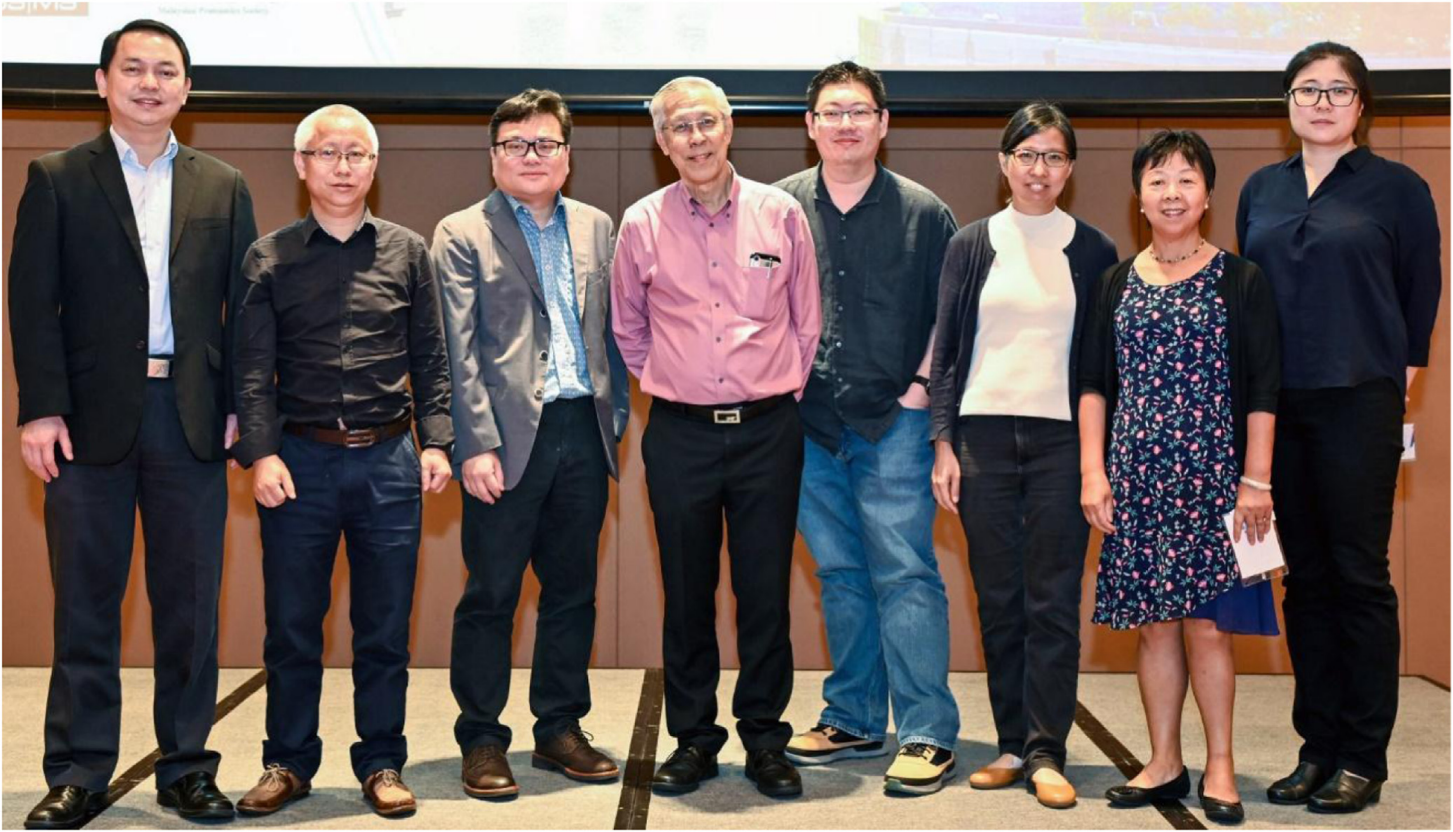
**Picture of some of the AOHUPO/AOAPO congress organizing committee members.** Qingsong Lin, Xuezhi Bi, Teck Yew Low, Maxey Chung, Qifeng Lin, Yee Jiun Kok, Yulan Wang, and Wei Wu. AOHUPO, Asia Oceania Human Proteomics Organization; AOAPO, Asia Oceania Agricultural Proteomics Organization.

After the closing ceremony, a by-invitation-only workshop was conducted by the π-HuB project team led by Prof. Fuchu He from the CNHUPO. During this workshop, five principal investigators from the π-HuB project, that is, Ruijun Tian, Jing Yang, Tiannan Guo, Ying Jiang, and Yunping Zhu presented their works, ranging from the state-of-the-art robotic and MS facilities in Guangzhou, high performance computing and AI infrastructure, to clinical proteomics applications. After dinner, each invitee was given 5 to 10 min to present their work and institutions. The presentations were followed by intense Q & A sessions and discussion.

## Postcongress Workshop (May 11th 2023)

The postcongress workshop was organized by the HUPO Education and Training Committee and was conducted in the National University of Singapore. The theme for this workshop was “Emerging Proteomics Technologies in the age of AI.” This one-day workshop was attended by 39 researchers and comprised five lectures that were delivered by Prof. Ruijun Tian, Prof. Limsoon Wong, Prof. Wilson Goh, Prof. Frederico Torta, and Prof. Tiannan Guo, covering topics such as highly sensitive proteomics-driven precision medicine, protein function prediction with machine learning, novel imputation methods for missing proteins, lipidomics, and the future of proteomics. During lunch time, participants also had the opportunity to have a guided tour to the Protein and Proteomics Center, as well as Singapore Lipidomics Incubator located within National University of Singapore. Furthermore, instrument vendors were also invited to share their latest product development.

## Future Perspectives

The AOHUPO congress has been held every 2 years since its inception in 2002. Traditionally, the host city and society(ies) for the next congress are announced at the closing of the current congress. However, this year there were three high-quality expressions of interest to host the 2025 congress. While these proposals are assessed during 2023, we are sure that members of the many AO regional societies already anticipate another highly successful congress that will be held in 2025. In the meantime, planning has already begun on AOHUPO events for 2024, which includes the third OES, an initiative borne of the Covid-19 pandemic but sure to remain a regular feature on the AOHUPO calendar for years to come. Going forward, the vision of AOHUPO is to hold congress and OES events on alternate years, which will accommodate at least one event each year to bring the community together to share, discuss, and celebrate proteomics and related ‘omics research across the AO region.

## Conflict of interest

The authors declare no competing interests.

## References

[1] Low T.Y., Chen Y.J., Ishihama Y., Chung M.C.M., Cordwell S., Poon T.C.W. (2022). The second Asia-Oceania human proteome organization (AOHUPO) online education series on the renaissance of clinical proteomics: biomarkers, imaging and therapeutics. Mol. Cell Proteomics.

